# Buffalo Horn Sign – A New Finding on MRI for Meniscal Bucket-Handle Tears

**DOI:** 10.1055/s-0045-1809336

**Published:** 2025-06-23

**Authors:** Rafael R. Pereira, João Cabral, João Janeiro, José Padín, Joaquim Soares do Brito, Rodrigo A. Goes

**Affiliations:** 1 Orthopedics Service, Unidade Local de Saúde Santa Maria, Lisbon, Portugal; 2 Knee and Ankle Surgery Unit, Centro de Ortopedia e Traumatologia, Hospital CUF Descobertas, Lisbon, Portugal; 3 Radiology Service, Unidade Local de Saúde Santa Maria, Lisbon, Portugal; 4 School of Medicine, Universidade de Lisboa, Lisbon, Portugal

**Keywords:** diagnostic imaging, knee, magnetic resonance imaging, tibial meniscus injuries, diagnóstico por imagem, imagem por ressonância magnética, joelho, lesões do menisco tibial

## Abstract

**Objective:**

To describe a new sign on magnetic resonance imaging (MRI) axial images of patients with bucket-handle meniscal tears.

**Methods:**

Of 610 consecutive patients with a surgical diagnosis of meniscal tear, those with a bucket-handle pattern were chosen, and 28 met the inclusion criteria. The most frequent mechanism was a twisting injury with or without a coronal stress (16 patients), and the injury was sports-related in 12 cases. All patients were symptomatic and had X-rays showing a preserved joint line. Next, their MRI examinations were analyzed.

**Results:**

The buffalo horn pattern was found in 13 patients (46.4%), occurring in either the medial or the lateral meniscus. It was the 3 ^rd^ most prevalent sign, after the fragment within the intercondylar notch ( *n* = 21; 75.0%) and the absent bow tie sign ( *n* = 17; 60.7%). We observed that it had a significant association with other signs of displaced meniscal handle. The sign was neither found on the healthy menisci, nor was affected by the occurrence of an anterior cruciate ligament tear.

**Conclusion:**

The buffalo horn is a new finding for displaced meniscal bucket-handle tears; it is easy to identify and relevant in the interpretation of axial MRI images. Its recognition is very important to determine the type of treatment and the surgical plan.

## Resumo

**Objetivo:**

Descrever um novo sinal em imagens axiais de ressonância magnética (RM) de pacientes com rupturas em alça de balde do menisco.

**Métodos:**

De 610 pacientes consecutivos com diagnóstico cirúrgico de ruptura do menisco, aqueles com padrão em alça de balde foram escolhidos, e 28 atenderam aos critérios de inclusão. O mecanismo de lesão mais frequente foi a torção com ou sem estresse coronal (16 pacientes). Além disso, a lesão foi relacionada ao esporte em 12 casos. Todos os pacientes eram sintomáticos e tinham radiografias que mostravam a preservação da linha articular. Em seguida, seus exames de RM foram analisados.

**Resultados:**

O padrão de chifre de búfalo foi encontrado em 13 pacientes (46,4%) no menisco medial ou lateral. Foi o 3 ^o^ sinal mais prevalente, depois do fragmento no interior da incisura intercondilar ( *n* = 21; 75,0%) e da ausência do sinal da gravata borboleta ( *n* = 17; 60,7%). Observamos uma associação significativa a outros sinais de deslocamento da alça do menisco. O sinal não foi encontrado em meniscos saudáveis, nem foi afetado pela ocorrência de ruptura do ligamento cruzado anterior.

**Conclusão:**

O chifre de búfalo é um novo achado para rupturas em alça de balde do menisco com deslocamento; é fácil de identificar e relevante na interpretação de imagens de RM de corte axial. Seu reconhecimento é muito importante para determinar o tipo de tratamento e o plano cirúrgico.

## Introduction

Menisci are crescent-shaped intracapsular fibrocartilagineous laminae with a role in load transmission, shock absorption, stability, lubrication, nutrient diffusion, sensory perception, and proprioception. 1 Meniscal tears are a common problem, with a reported incidence of ∼ 60 per 100 thousand inhabitants in the United States. 2 There are several patterns. Bucket-handle tears consist in a full-thickness longitudinal tear that propagates anteriorly and posteriorly, creating an inner fragment – the “handle” – that can displace into the intercondylar notch. 3 These lesions represent ∼ 10% of all tears; 3 4 they occur mainly in the medial meniscus, 4 5 but they can also affect the lateral meniscus. 6 7 8 Since surgical treatment is often required, the correct preoperative diagnosis is important to optimize treatment and save meniscal tissue. 9 Magnetic resonance imaging (MRI) is the gold-standard imaging method with an overall reported sensitivity of up to 90.0% and specificity of up to 89.0%. 10 11 12 13 On MRI, meniscal bucket-handle tears usually display some well-known signs, mainly in the coronal and sagittal sections: absent bow tie sign, 14 double posterior cruciate ligament (PCL) sign, 15 16 double anterior horn sign, 17 flipped meniscus sign, 18 disproportional posterior horn sign, 19 double anterior cruciate ligament (ACL) sign, 20 triple PCL sign, 7 triple cruciate sign, 8 quadruple cruciate sign, 6 and the presence of a fragment within the intercondylar notch 4 ( Appendix A ). The reported sensitivity and specificity for the diagnosis of meniscal bucket-handle tears varies in a wide range, from 64.0 to 93.0% 4 5 21 and from 64.0 to 100% 10 22 respectively – but it improves if more signs are known. 5 22

**Appendix A:** Definition of the characteristic findings on magnetic resonance imaging of meniscal bucket-handle tears

Authors	Sign	Definition
Weiss et al., 1991 15 Singson et al., 1991 16	Double PCL	A low signal band anterior and parallel to the posterior cruciate ligament in sagittal images.
Haramati et al., 1993 18	Flipped meniscus	An abnormally-enlarged anterior meniscal horn (> 6 mm).
Wright et al., 1995 4	Intercondylar fragment	A band-like area of low signal intensity within the notch but not appearing on the same slice as the PCL.
Helms et al., 1998 14	Sign of absence of bow tie	The occurrence of only one or no meniscal body segment in consecutive MRI sagittal images.
Ruff et al., 1998 17	Double anterior horn	The presence of two triangles not vertically juxtaposed but located next to another in the same horizontal plane in a sagittal section appearing like two anterior horns of the meniscus.
Chen et al., 2001 19	Disproportional posterior horn	Posterior horn in the central section larger than that in the peripheral section on sagittal MRI images.
Bugnone et al., 2005 6	Quadruple cruciate sign	Four structures in intercondylar notch observed in consecutive coronal sections: both displaced fragments of torn menisci, the stump of torn ACL and the intact PCLç.
Bui-Mansfield et al., 2006 20	Double ACL	The presence of the fragment immediately posterior to the ACL.
Kakel et al., 2010 7	Triple PCL	The presence of an intact PCL and two displaced fragments in the intercondylar notch from the two bucket-handle tears on sagittal view in an ACL-deficient knee.
Rao et al., 2012 23	V sign	The “V” is seen at the junction of the displaced fragment (handle), as it forms a right angle with the meniscus, which is in place.
Sales et al., 2021 8	Triple cruciate sign	Three structures in intercondylar notch observed in coronal sections: both displaced fragments of torn menisci and the intact PCL.
Barrie, 1979 25	Parameniscal cyst	A fluid collection in intimate relation with the meniscus either by a direct contact or a fluid track.
Gale et al., 1999 26	Meniscal extrusion	Quantified in the coronal image at its greatest value and was considered when the peripheral margin of the meniscus extends 3 mm or more beyond the edge of the tibial plateau.
Kaplan et al., 1999 27	Subchondral marrow edema	Nonlinear edema with no clearly-defined margin.
Kolman et al., 2004 24	Joint effusion	An anteroposterior measurement of 10 mm or more in the lateral suprapatelar pouch was considered abnormal.
Bergin et al., 2008 28	Linear subchondral marrow edema	Well-demarcated edema parallel to the articular surface and fewer than 5 mm deep.

**Abbreviations:** ACL, anterior cruciate ligament; MRI, magnetic resonance imaging; PCL, posterior cruciate ligament.

To the best of our knowledge, there was only one sign previously described in MRI axial view: the V sign. 23 The present paper aims to report a new sign to be identified in that view, which is perceived as a buffalo horn and appears in patients with meniscal bucket-handle tears. The sensitivity of this finding will be determined and compared with the presence of the other already-known signs.

## Materials and Methods

### Compliance with Ethical Standards

The current study follows the ethical standards of the institutional Research Committee and of the 1964 Declaration of Helsinki and its later amendments or comparable ethical standards. It received approval n. 215/22 from the Ethics Board of Centro Acadêmico Médico de Lisboa. Informed consent was exempted as long as personal data protection standards were met, but additional written informed consent was obtained from all patients for whom MRI scans are included in the present article.

This is a retrospective study based on MRI scans of patients diagnosed during surgery with meniscal bucket-handle tears, disregarding age, sex, mechanism, the time between trauma and MRI and between trauma and surgery, treatment, surgeon, ACL injury, and affected knee or meniscus.

**Fig. 1:**
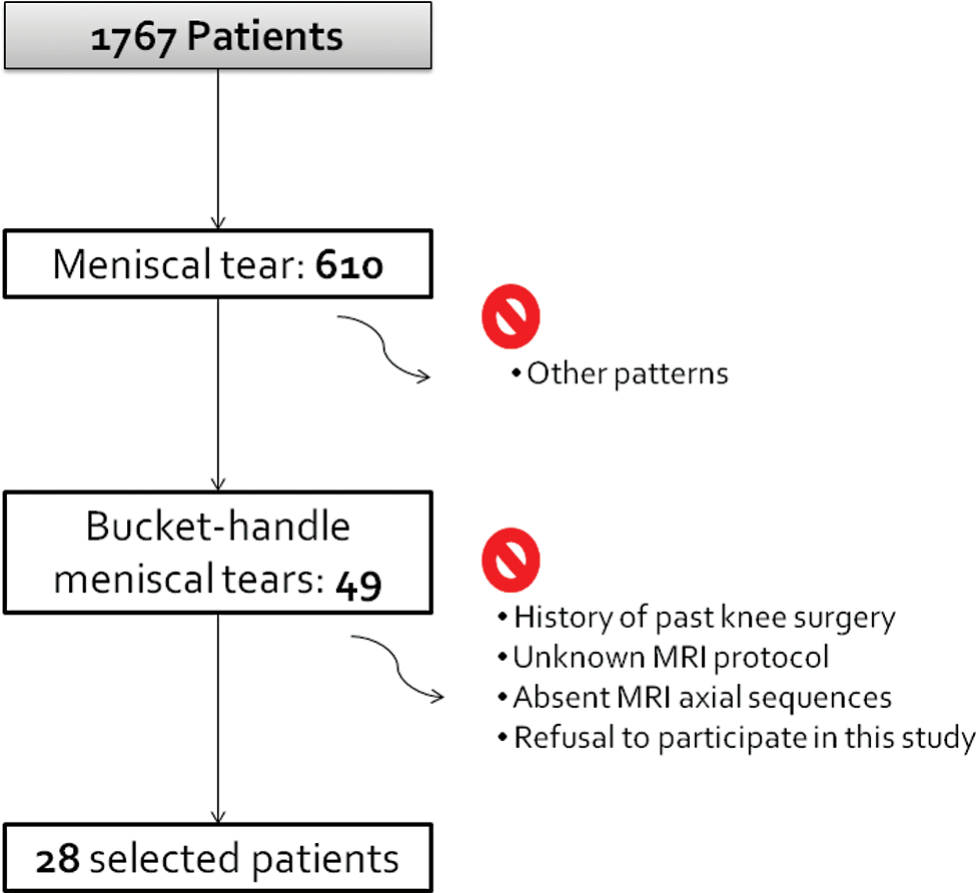
Flowchart illustrating the search strategy and selection criteria.

A consecutive sample of 1,767 patients operated at the Orthopedics Service of a university hospital for any knee pathology between 2012 and 2021 was obtained ( Fig. 1 **)** . This period was chosen because contains the highest number of patients available at our institution due to the limited computerization of medical files in the previous years. Only 610 patients had surgically proven meniscal tears, and 49 showed a bucket-handle pattern. Next, the patients with the following criteria were excluded: history of knee surgery, other patterns of meniscal tears, absent meniscal tear, unknown MRI protocol, absent axial sequences or menisci not acquired, and refusal to participate in the research. The collected sample was of 28 patients, 22 men and 6 women, with a mean age of 34.2 (± 14.0; range: 9–63) years. The most frequent mechanism of injury reported by patients was torsion with or without coronal load (16 patients). Four patients experienced the injury after a knee flexion or a squat. Two cases suffered a fall from their height, and one described a complex trauma during surf practice. Five patients could not identify any trauma.

Twelve of these injuries were sports-related. Every patient showed, at some degree, knee pain, joint effusion, a sensation of locking or looseness, or loss of extension. All the X-rays showed a preserved joint line.

In total, 24 MRI scans were performed at the hospital where this research was conducted, and 4 were performed elsewhere, but with a similar protocol. Seventeen were performed with Philips Intera 1.5 T scanner, and 11 were performed with the Philips Achieva 3.0 T scanner. The receive-only specific knee coil provided by the manufacturer for each scanner was used. The standard acquisitions are summarized in Table 1 , and they include: a coronal T1, T2 fast field echo (FFE) and short tau inversion recovery (STIR); a sagittal proton density (PD) with and without fat suppression; and an axial T2 FFE. Some exams had an additional coronal T2 spectral attenuated inversion recovery (SPAIR) and an axial SPAIR. A slightly different protocol for the Intera scanner was used to assess the pediatric patient, using a slice of 3 mm and a gap of 0.3 mm.

**Table 1:** Magnetic resonance imaging acquisition protocols

*Philips Intera (1.5 T)*							
	Matrix	RT/ET (ms)	Slice (mm)	Gap (mm)	Average	FOV (mm)	FA (°)
Coronal							
T1	400 × 300	500/22	3.5	0.35	2	180	90
T2 FFE	300 × 250	500/14	3.5	0.35	2	180	25
STIR	250 × 200	5,000/80	3.5	0.35	3	180	–
T2 SPAIR	300 × 250	3,000/60	3.5	0.35	2	180	90
Sagittal							
PD	300 × 250	2,500/8	3.5	0.35	2	180	90
PD SPAIR	250 × 250	3,000/30	3.5	0.35	3	180	90
T2	300 × 250	2,500/120	3.5	0.35	2	180	90
Axial							
T2 FFE	250 × 200	600/14	3.5	0.35	2	180	25
SPAIR	250 × 200	3,000/30	3.5	0.35	4	180	90
***Philips Achieva (3 T)***							
Coronal							
T1	400 × 350	600/20	3	0.3	2	180	90
T2 FFE	400 × 300	450/12	3	0.3	2	180	20
STIR	250 × 200	4,000/80	3.5	0.35	2	180	–
T2 SPAIR	300 × 250	4,000/65	3	0.3	2	180	90
Sagittal							
PD	400 × 300	7,000/9	3	0.3	1	180	90
PD SPAIR	300 × 300	5,000/30	3	0.3	2	180	90
T2	400 × 300	7,000/140	3	0.3	1	180	90
Axial							
T2 FFE	300 × 250	500/12	3	0.3	2	180	20
SPAIR	300 × 250	3,800/65	3	0.3	2	180	90

**Abbreviations:** ET, echo time; FA, flip angle; FFE, fast field echo; FOV, field of view; PD, proton density; RT, repetition time; SPAIR, spectral attenuated inversion recovery; STIR, short tau inversion recovery.

All surgeries were performed by senior knee surgeons, with fellowship training. Meniscal bucket-handle tear was defined as “a longitudinal tear with central migration of the ‘inner’ handle fragment.” 3

**Fig. 2:**
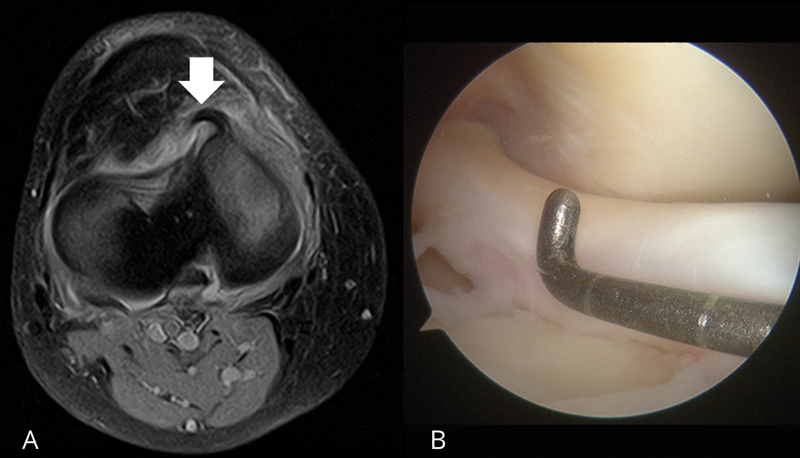
( **A** ) Preoperative MRI scan of a 54 year-old male patient with a surgically-reported bucket-handle tear of the medial meniscus of the right knee showing the buffalo horn sign (arrow) in the axial cross-section. This finding is a low signal intensity area projecting from the anterior border of the medial tibial plateau, resembling a horn. ( **B** ) Right knee arthroscopy using an anterolateral portal, showing the meniscal fragment of the same patient displaced anteriorly to the femoral condyle. Axial spectral attenuated inversion recovery (SPAIR) sequence: matrix, 250 × 200; repetition time/echo time (RT/ET), 3,000/30 milliseconds; slice, 3,5 mm; gap, 0,35; average, 4; field of view (FOV), 180 mm; flip angle (FA), 90°.

**Fig. 3:**
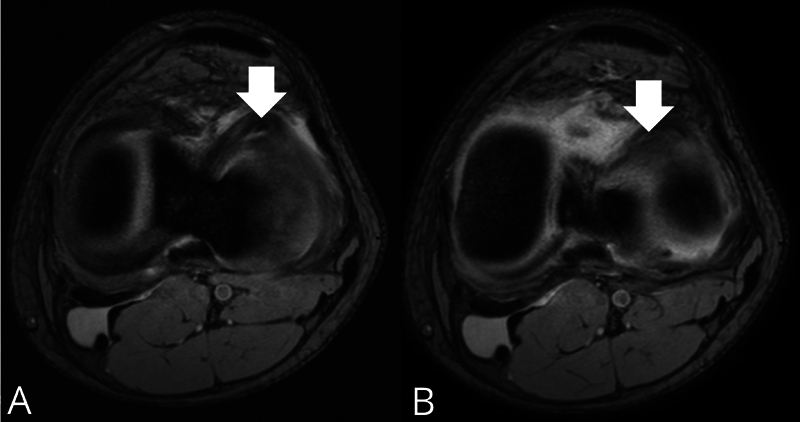
Preoperative MRI scan of a 17 year-old male subject with a surgically-reported bucket-handle tear of the lateral meniscus of the left knee. ( **A,B** ) Axial cross-section in T2 fast field echo (FFE) showing the buffalo horn sign (arrow) in lateral meniscus appearing as a low signal intensity horn-shaped band lying parallel to the anterior border of lateral tibial plateau in two consecutive axial images. Axial T2 FFE: matrix, 300 × 250; RT/ET, 500/12 milliseconds; slice, 3 mm; FOV, 180 mm; FA, 20°.

A senior knee surgeon and an orthopedics resident were instructed on MRI interpretation of bucket-handle tears and then prospectively and blindly assessed the images. Consensus was established by the senior musculoskeletal radiologist. Each MRI scan was assessed for the presence of an absent bow tie sign, 14 double PCL sign, 15 16 double anterior horn sign, 17 flipped meniscus sign, 18 disproportional posterior horn sign, 19 double ACL sign, 20 triple PCL sign, 7 triple cruciate sign, 8 quadruple cruciate sign, 6 and the presence of a fragment within the intercondylar notch. 4 When in cross-section, the V sign 23 and the new sign were recorded. The buffalo horn sign is the presence of a low signal intensity area projecting from the anterior border of the medial tibial plateau, resembling a horn which can be appreciated in the MRI axial view, as demonstrated in Fig. 2 . If the lateral meniscus is affected, the sign appears like a low-intensity horn-shaped band lying parallel to the anterior border of the lateral tibial plateau in two consecutive axial images ( Fig. 3 ). The other definitions used are reviewed in Appendix A . The presence of joint effusion, 24 parameniscal cyst, 25 meniscal extrusion, 26 bone marrow edema and its location 27 28 were also recorded. The cases with MRI suspicion of hemibucket-handle tear of the meniscus were counted. 29 Surgical evidence of rupture of the ACL was reported.

A literature search for the terms and expressions *menisci* , *bucket handle* and *MRI scan* was conducted on the PubMed search engine. Only studies written in English and involving human subjects were considered. No reports regarding a buffalo horn sign could be found.

### Statistical Analysis

For the statistical analysis, version 18.0 of the software PASW Statistics for Windows was used. Values of *p lower* than 0.05 were considered significant. The nominal variables were analyzed with the Chi-squared (χ ^2^ ) test or the Fisher's Exact test when > 20% of the cells had an expected count lower than 5.

## Results

The buffalo horn sign was found in 13 out of 28 cases (46.4%), which made it the third most prevalent sign, after the fragment within the intercondylar notch ( *n* = 21; 75.0%) and the absent bow tie sign ( *n* = 17; 60.7%). It occurred in 7/19 medial menisci (36.8%) ( Fig. 2 ) and 6/9 lateral menisci (66.7%) ( Fig. 3 ). If findings suggesting an anteriorly displaced handle were present ( *n* = 9), the new sign appeared in 7 cases (77.8%), 3 medial menisci (33.3%), and 4 lateral menisci (66.7%). If findings suggesting a posteriorly displaced fragment were present ( *n* = 4), the buffalo sign was observed in 2 patients (50.0%), 1 medial and 1 lateral meniscus. None of the aforementioned findings were statistically significant.

A significant association was observed between the new sign and a fragment within the intercondylar notch ( *p* = 0.01) and a double anterior horn sign ( *p* = 0.01) ( Table 2 ). There were no other statistically significant findings. The buffalo horn sign was observed regardless of an ACL tear ( Table 3 ).

**Table 2:** Frequency of the buffalo horn sign

Buffalo horn sign						
Sign	Present ( *n* = 13): *n* (%)	Absent ( *n* = 15): *n* (%)	Total ( *n* = 28): *n* (%)	OR (CI)	Statistical test	*p*
Intercondylar fragment	13 (100%)	8 (53%)	21 (75%)	**0.53** **(0.33–0.86)**	**FE**	**0.01**
Absent bow tie	10 (77%)	7 (47%)	17 (60%)	3.81 (0.74–19.67)	χ ^2 ^ = 2.67	0.10
V sign	6 (46%)	3 (20%)	9 (32%)	3.43 (0.65–18.22)	FE	0.23
Double PCL	6 (46%)	2 (13%)	8 (28%)	5.57 (0.88–35.27)	FE	0.10
Flipped meniscus	6 (46%)	2 (13%)	8 (29%)	5.57 (0.88–35.27)	FE	0.10
Double anterior horn	5 (39%)	0 (0%)	5 (18%)	**1.63** **(1.06–2.50)**	FE	**0.01**
Disproportional posterior horn	5 (39%)	2 (13%)	7 (25%)	4.06 (0.63–26.13)	FE	0.20
Double ACL	2 (15%)	0 (0%)	2 (7%)	1.18 (0.94–1.49)	FE	0.21
Indirect signs						
Joint effusion	10 (77%)	7 (47%)	17 (60%)	3.81 (0.74–19.66)	χ ^2 ^ = 2.67	0.10
Subchondral marrow edema	5 (39%)	4 (27%)	9 (32%)	1.72 (0.35–8.51)	FE	0.69
Linear subchondral marrow edema	3 (23%)	5 (33%)	8 (29%)	0.60 (0.11–3.21)	FE	0.69
MTP tibial plateau subchondral edema	3 (23%)	6 (40%)	9 (32%)	0.45 (0.09–2.35)	FE	0.44
Meniscal extrusion	2 (15%)	5 (33%)	7 (25%)	0.36 (0.06–2.31)	FE	0.40
Parameniscal cyst	0 (0%)	2 (13%)	2 (8%)	0.88 (0.71–1.06)	FE	0.48
Other conditions						
ACL rupture	5 (39%)	6 (40%)	11 (39%)	0.94 (0.21–4.29)	χ ^2 ^ = 0.01	0.93
Hemibucket handle	0 (0%)	3 (20%)	3 (11%)	0.80 (0.62–1.03)	FE	0.23

**Abbreviations:** χ ^2^ , Chi-squared test; ACL, anterior cruciate ligament; FE, Fisher's exact test; MTP, medial tibial plateau; OR, odds ratio; PCL, posterior cruciate ligament.

**Notes:** An association was observed between fragment within intercondylar notch and double anterior horn with the new reported sign. The χ ^2^ and FE tests were performed. Statistically significant *p* -values are in bold.

**Table 3:** Anterior cruciate ligament tear and the frequency of signs of bucket-handle tear

ACL tear						
Sign	Present ( *n* = 11): *n* (%)	Absent ( *n* = 17): *n* (%)	Total ( *n* = 28): *n* (%)	OR (CI)	Statistical test	*p*
Intercondylar fragment	7 (64%)	14 (82%)	21 (75%)	0.38 (0.07–2.16)	FE	0.38
Absent bow tie	8 (73%)	9 (53%)	17 (60%)	2.37 (0.46–12.14)	FE	0.44
Buffalo horn	5 (46%)	8 (47%)	13 (46%)	0.94 (0.21–4.29)	χ ^2 ^ = 0.01	0.93
V sign	4 (36%)	5 (29%)	9 (32%)	1.37 (0.27–6.87)	FE	1.00
Double PCL	3 (27%)	5 (29%)	8 (28%)	0.90 (0.17–4.87)	FE	1.00
Flipped meniscus	2 (18%)	6 (35%)	8 (29%)	0.41 (0.07–2.53)	FE	0.42
Disproportional posterior horn	4 (36%)	3 (18%)	7 (25%)	2.67 (0.46–15.35)	FE	0.38
Double anterior horn	4 (36%)	1 (6%)	5 (18%)	9.14 (0.86–97.27)	FE	0.06
Double ACL	0 (0%)	2 (12%)	2 (8%)	0.88 (0.74–1.05)	FE	0.51
Indirect signs						
Joint effusion	7 (64%)	10 (59%)	17 (60%)	1.22 (0.26–5.85)	FE	1.00
Subchondral marrow edema	6 (65%)	3 (18%)	9 (32%)	5.60 (1.01–31.32)	FE	0.38
PTM subchondral edema	6 (65%)	3 (18%)	9 (32%)	5.6 (1.00–31.32)	FE	0.10
Linear subchondral marrow edema	2 (18%)	6 (25%)	8 (29%)	0.41 (0.07–2.53)	FE	0.42
Meniscal extrusion	3 (27%)	4 (23%)	7 (25%)	1.22 (0.22–6.92)	FE	1.00
Parameniscal cyst	0 (0%)	2 (12%)	2 (8%)	0.8 (0.74–1.05)	FE	0.51
Other conditions						
Hemibucket handle	0 (0%)	3 (18%)	3 (11%)	0.8 (0.66–1.03)	FE	0.26

**Abbreviations:** χ ^2^ , Chi-squared test; ACL, anterior cruciate ligament; FE, Fisher's exact test; MTP, medial tibial plateau; OR, odds ratio; PCL, posterior cruciate ligament.

**Notas:** There were no differences in terms of the prevalence of the findings between the groups with or without ACL tear. The χ ^2^ and FE tests were performed.

Looking at the findings of the axial sections, the V sign was identified in 9 cases, a sensitivity of 32.1%. Of these nine patients, three presented only the V sign, and six also presented the buffalo horn sign. Of 13 patients with the latter, 7 had only the buffalo horn sign, but not the V sign. None of those signs appeared as the only sign for the tear, and all cases had the fragment within the intercondylar notch and the sign of absence of the bowtie.

The medial meniscus was torn in 19 cases (67.8%) and the lateral, in 9 (32.2%) cases. Three cases exhibit the hemibucket-handle pattern, 27 which could not be confirmed through the surgical records, and none of these patients presented the buffalo horn sign. Six MRI scans missed the diagnosis of bucket-handle tear, but all presented at least one indirect sign. The new sign was never found on an unaffected meniscus.

## Discussion

**Fig. 4:**
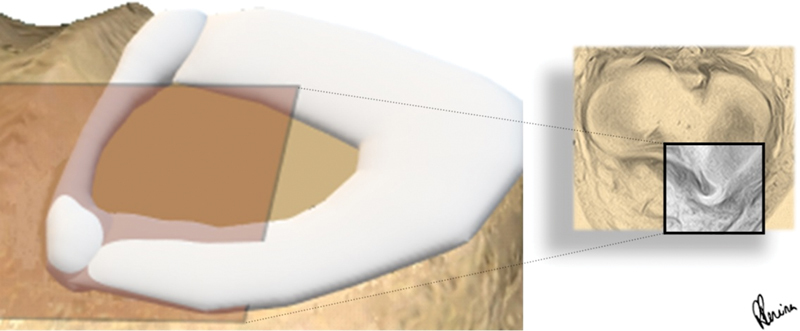
The dislocated bucket-handle fragment of the medial meniscus of a right knee is sliced in axial section (orange box), appearing as a horn.

The most relevant finding of the present study was that the buffalo horn could be a new sign to diagnose meniscal bucket-handle tears. It is useful for both menisci in fragments displaced anterior 18 20 or posterior 19 dislocated fragments. It was found in 7/19 medial menisci (36.8%) and 6/9 lateral menisci (66.7%), without statistically significant differences. All cases with a double anterior horn presented the buffalo horn sign ( *p* = 0.01). In 8/28 (28.6%) cases with a flipped meniscus, the proposed sign appeared in 6 (75.0%). Both patients with double ACL presented it (100%). The studied finding also appeared in 2/4 disproportional posterior horn cases. Additionally, all patients with the new finding presented a fragment within the intercondylar notch ( *p* = 0.01), and the new sign never appeared alone, but always in the presence of other signs for a displaced fragment. Thus, one can conclude that the buffalo horn sign means a displaced handle of a meniscal tear, and the horn appears when the axial section bisects it ( Fig. 4 ).

**Fig. 5:**
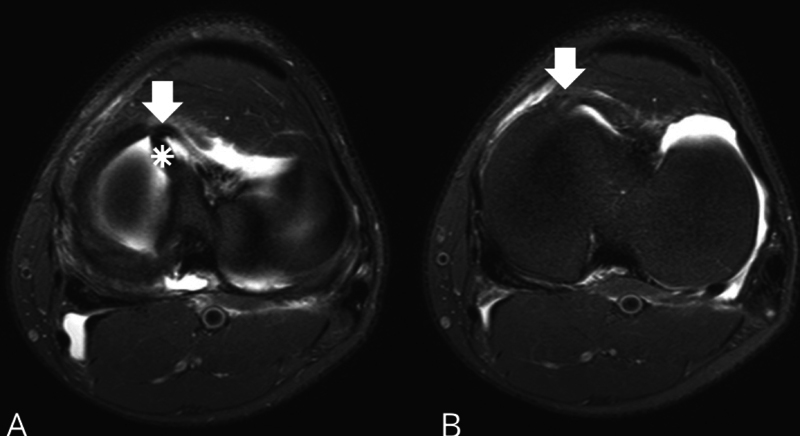
Cross-sections of a preoperative MRI scan of a 27 year-old male patient with a bucket-handle tear of the medial meniscus of the left knee. ( **A** ) The V sign (*) and the buffalo horn sign (arrow) are visible. ( **B** ) In the following section, only the buffalo horn sign (arrow) is found. Axial T2 SPAIR: matrix, 300 × 250; RT/ET, 3800/65 milliseconds; slice, 3 mm; gap, 0.3; average, 2; FOV, 180 mm; FA, 90°.

This new finding was observed because the assessment of cross-sectional images is gaining importance in clinical practice, and there was only one sign previously described for this condition in these images: the V sign. 23 Despite being close to each other, these two findings are not the same. The buffalo horn uses a sequence of images, appears anterior, and does not need to have the handle and the meniscus in the same section. Seven patients had the buffalo horn sign but not the V sign, and 3 had the latter but not the former. Six out of nine patients had both findings simultaneously ( Fig. 5 ). In this sample, the V sign had 32.1% of sensitivity, which is lower than the rate previously reported by Rao et al., 23 of 72.0%, and it is also lower than the sensitivity of the buffalo horn sign in the sample of the present study (46.4%).

The overall sensitivity of MRI in the current study was low, corresponding to the lower half of the previously-reported range of 64 to 93%. 4 5 12 13 21 22 In total, 6 out of 28 MRI scans missed any sign of a meniscal bucket-handle tear, which translates to an overall sensitivity of only 78.6%. This fact may explain the low sensitivity of the buffalo horn sign (46.4%), even though it was the third most relevant sign, with higher sensitivity than the other 6 widely-spread findings. Its sensitivity was surpassed by the fragment within the intercondylar notch (21/28; 75.0%) and the absent bow tie sign (17/28; 60.7%). That is consistent with the reported sensitivity by Dorsay and Helms for the fragment within the intercondylar notch of 76.7%, 22 but is lower than the 88.4% described for the absence of the bowtie sign in the same study. In the present research, we do not intend to calculate either the specificity or reproducibility of the sign, but we never found the buffalo horn on an unaffected meniscus; moreover, the observation of the sign requires a displaced fragment to show up. Therefore, we believe that it is specific. Additionally, the proposed definition and the appearance of the buffalo horn sign are easy to spot, maybe more than the fragment within the intercondylar notch or the absence of the bow tie, whose definitions are vague.

The present study has several limitations. The sample is small due to the obstacles faced, such as the limited computerization of medical files before 2012 and a low volume of treated patients. This prevented us from obtaining a larger sample, but we believe that it does not threaten the aim of the current paper. The unaffected menisci were used as controls, and the time elapsed from the trauma to the MRI scan and surgery may be a source of bias. The patients were chosen based on a surgical diagnosis, which did not enable the performance of a test for the specificity. The reliability of the sign was not calculated. The sample gathers different magnetic field intensities, but Van Dyck et al. 30 showed, in a controlled prospective study, that diagnostic accuracy of 3.0 T for meniscal and ACL tears is not significantly higher than 1.5 T, so it seems reasonable to believe that this issue does not compromise the identification of a new sign. Knees with ACL deficiency (acute or chronic; 11/28; 39.3%) were studied aside from ACL-competent knees, but there are controversies regarding their effect on the accuracy of MRI. 10 12 Although all the surgeries were performed by experienced knee surgeons working in the same department, the surgical reports had not been standardized, and some missed the subtype classification of the bucket-handle tear.

## Conclusion

In conclusion, the buffalo horn sign could be present in the medial and lateral torn menisci, with and without ACL tear. A dislocated fragment is the cause of the sign, so it is statistically associated with the fragment within the intercondylar notch or the double anterior horn sign. Recognizing this situation is very important to determine the type of treatment and to be able to plan the surgery. Moreover, we believe that this finding is easy to identify, and it is specific for bucket-handle tears, because it was not found on unaffected menisci.

Herein, the buffalo horn sign appears as a new finding that can be relevant to diagnose meniscal bucket-handle tears using axial MR imaging.

## References

[1] Masouros S D McDermott I D Amis A A Bull A MJ Biomechanics of the meniscus-meniscal ligament construct of the knee Knee Surg Sports Traumatol Arthrosc 2008 16 12 1121 1132 10.1007/s00167-008-0616-9 18802689

[2] Baker B E Peckham A C Pupparo F Sanborn J C Review of meniscal injury and associated sports Am J Sports Med 1985 13 01 1 4 10.1177/036354658501300101 3838420

[3] Shakespeare D T Rigby H S The bucket-handle tear of the meniscus. A clinical and arthrographic study J Bone Joint Surg Br 1983 65 04 383 387 10.1302/0301-620X.65B4.6874707 6874707

[4] Wright D H De Smet A A Norris M Bucket-handle tears of the medial and lateral menisci of the knee: value of MR imaging in detecting displaced fragments AJR Am J Roentgenol 1995 165 03 621 625 10.2214/ajr.165.3.7645481 7645481

[5] Ververidis A N Verettas D A Kazakos K J Tilkeridis C E Chatzipapas C N Meniscal bucket handle tears: a retrospective study of arthroscopy and the relation to MRI Knee Surg Sports Traumatol Arthrosc 2006 14 04 343 349 10.1007/s00167-005-0678-x 16163557

[6] Bugnone A N Ramnath R R Davis S B Sedaros R The quadruple cruciate sign of simultaneous bicompartmental medial and lateral bucket-handle meniscal tears Skeletal Radiol 2005 34 11 740 744 10.1007/s00256-005-0915-x 15895223

[7] Kakel R Russell R VanHeerden P The triple PCL sign: bucket handle tears of both medial and lateral menisci in a chronically ACL-deficient knee Orthopedics 2010 33 10 772 10.3928/01477447-20100826-22 20954659

[8] Sales E Gupta S Daines B Baker A Landgrabe M Zeini I M Bicompartmental Bucket Handle Meniscal Tear with Chronic ACL Deficiency Causing a Rare Triple PCL and Triple Cruciate Sign: A Case Report JBJS Case Connect 2021 11 02 e20.00694 10.2106/JBJS.CC.20.00694 33974600

[9] Goes R A Cavalcanti A S Campos A LS Cardoso RdF Coelho O N McCormack R G Prediction of reparability of meniscal tears in athletes using magnetic resonance J Biol Regul Homeost Agents 2020 34(4, Suppl. 3)153 162 33261272

[10] Naranje S Mittal R Nag H Sharma R Arthroscopic and magnetic resonance imaging evaluation of meniscus lesions in the chronic anterior cruciate ligament-deficient knee Arthroscopy 2008 24 09 1045 1051 10.1016/j.arthro.2008.03.008 18760213

[11] De Smet A A Tuite M J Norris M A Swan J S MR diagnosis of meniscal tears: analysis of causes of errors AJR Am J Roentgenol 1994 163 06 1419 1423 10.2214/ajr.163.6.7992739 7992739

[12] Figueiredo S Sa Castelo L Pereira A D Machado L Silva J A Sa A Use of MRI by radiologists and orthopaedic surgeons to detect intra-articular injuries of the knee Rev Bras Ortop 2017 53 01 28 32 10.1016/j.rboe.2016.12.013 29367903 PMC5771798

[13] Orlando N Júnior Leão MGdS Oliveira N HD Diagnosis of knee injuries: comparison of the physical examination and magnetic resonance imaging with the findings from arthroscopy Rev Bras Ortop 2015 50 06 712 719 10.1016/j.rboe.2015.10.007 27218085 PMC4867911

[14] Helms C A Laorr A Cannon W D Jr The absent bow tie sign in bucket-handle tears of the menisci in the knee AJR Am J Roentgenol 1998 170 01 57 61 10.2214/ajr.170.1.9423600 9423600

[15] Weiss K L Morehouse H T Levy I M Sagittal MR images of the knee: a low-signal band parallel to the posterior cruciate ligament caused by a displaced bucket-handle tear AJR Am J Roentgenol 1991 156 01 117 119 10.2214/ajr.156.1.1898543 1898543

[16] Singson R D Feldman F Staron R Kiernan H MR imaging of displaced bucket-handle tear of the medial meniscus AJR Am J Roentgenol 1991 156 01 121 124 10.2214/ajr.156.1.1898544 1898544

[17] Ruff C Weingardt J P Russ P D Kilcoyne R F MR imaging patterns of displaced meniscus injuries of the knee AJR Am J Roentgenol 1998 170 01 63 67 10.2214/ajr.170.1.9423601 9423601

[18] Haramati N Staron R B Rubin S Shreck E H Feldman F Kiernan H The flipped meniscus sign Skeletal Radiol 1993 22 04 273 277 10.1007/BF00197673 8316871

[19] Chen H C Hsu C Y Shih T T Huang K M Li Y W MR imaging of displaced meniscal tears of the knee. Importance of a “disproportional posterior horn sign” Acta Radiol 2001 42 04 417 421 10.1080/028418501127346918 11442468

[20] Bui-Mansfield L T DeWitt R M Magnetic resonance imaging appearance of a double anterior cruciate ligament associated with a displaced tear of the lateral meniscus J Comput Assist Tomogr 2006 30 02 327 332 10.1097/00004728-200603000-00032 16628058

[21] Magee T H Hinson G W MRI of meniscal bucket-handle tears Skeletal Radiol 1998 27 09 495 499 10.1007/s002560050426 9809878

[22] Dorsay T A Helms C A Bucket-handle meniscal tears of the knee: sensitivity and specificity of MRI signs Skeletal Radiol 2003 32 05 266 272 10.1007/s00256-002-0617-6 12719929

[23] Rao N Patel Y Opsha O Chen Q Owen J Eisemon E Use of the V-sign in the diagnosis of bucket-handle meniscal tear of the knee Skeletal Radiol 2012 41 03 293 297 10.1007/s00256-011-1181-8 21656136

[24] Kolman B H Daffner R H Sciulli R L Soehnlen M W Correlation of joint fluid and internal derangement on knee MRI Skeletal Radiol 2004 33 02 91 95 10.1007/s00256-003-0707-0 14614577

[25] Barrie H J The pathogenesis and significance of menisceal cysts J Bone Joint Surg Br 1979 61-B 02 184 189 10.1302/0301-620X.61B2.582035 582035

[26] Gale D R Chaisson C E Totterman S M Schwartz R K Gale M E Felson D Meniscal subluxation: association with osteoarthritis and joint space narrowing Osteoarthritis Cartilage 1999 7 06 526 532 10.1053/joca.1999.0256 10558850

[27] Kaplan P A Gehl R H Dussault R G Anderson M W Diduch D R Bone contusions of the posterior lip of the medial tibial plateau (contrecoup injury) and associated internal derangements of the knee at MR imaging Radiology 1999 211 03 747 753 10.1148/radiology.211.3.r99jn30747 10352601

[28] Bergin D Hochberg H Zoga A C Qazi N Parker L Morrison W B Indirect soft-tissue and osseous signs on knee MRI of surgically proven meniscal tears AJR Am J Roentgenol 2008 191 01 86 92 10.2214/AJR.07.3313 18562729

[29] Engstrom B I Vinson E N Taylor D C Garrett W E Jr Helms C A Hemi-bucket-handle tears of the meniscus: appearance on MRI and potential surgical implications Skeletal Radiol 2012 41 08 933 938 10.1007/s00256-011-1321-1 22080362

[30] Van Dyck P Vanhoenacker F M Lambrecht V Wouters K Gielen J L Dossche L Parizel P M Prospective comparison of 1.5 and 3.0-T MRI for evaluating the knee menisci and ACL J Bone Joint Surg Am 2013 95 10 916 924 10.2106/JBJS.L.01195 23677359

